# Associations Between Sleep Duration and Cognitive Function Among Older Adults: Cross-Sectional Study

**DOI:** 10.2196/72886

**Published:** 2025-09-08

**Authors:** Guolin Guo, Yanling Jiang, Jianteng Dong, Xu Zhao, Xiaofan Lai, Xiumei Wang, Hongguo Rong, Jian Li

**Affiliations:** 1 School of Traditional Chinese Medicine Beijing University of Chinese Medicine Beijing China; 2 School of humanities Beijing University of Chinese Medicine Beijing China; 3 Center for Evidence-Based Chinese Medicine Beijing University of Chinese Medicine Beijing China

**Keywords:** sleep, cognitive function, aging, China Health and Retirement Longitudinal Study, CHARLS

## Abstract

**Background:**

Sleep duration plays a crucial role in cognitive health and is closely linked to cognitive decline. However, the relationship between sleep duration and cognitive function in the Chinese population remains poorly understood.

**Objective:**

This study aims to evaluate the association between sleep duration and cognitive function among middle-aged and older adults in China.

**Methods:**

Using data from 15,526 participants in the 2020 China Health and Retirement Longitudinal Study, we used 3 composite indicators, encompassing episodic memory, mental acuity, and overall cognitive function to evaluate cognitive performance. Sleep duration per night, self-reported through face-to-face interviews, was also obtained. Adjustments were made using multiple generalized linear regression models, accounting for demographic, lifestyle, and health-related covariates.

**Results:**

Among the 15,526 respondents analyzed, 53.02% (8232/15,526) were female and 46.98% (7294/15,526) were male, with an average age of 61.5 (SD 9.27) years. Those reporting sleep durations of 4 hours or less (β=−1.85, 95% CI −2.07 to −1.62), 5 hours (β=−0.55, 95% CI −0.78 to −0.33; *P*<.001), 9 hours (β=−1.78, 95% CI −2.17 to −1.39), and 10 hours or more (β=−3.01, 95% CI −3.39 to −2.63) per night had a significant negative relationship with cognitive function. In the adjusted model, the negative impact of long sleep (≥10 hours) on overall cognitive function became more pronounced (β=−3.01, 95% CI −3.39 to −2.63; *P*<.001), followed closely by extremely short sleep (≤4 hours; β=−1.85, 95% CI −2.07 to −1.62; *P*<.001).

**Conclusions:**

This study reveals an inverted U-shaped relationship between sleep duration and global cognitive decline, indicating that cognitive function should be closely monitored in individuals with both short and long sleep durations. Consequently, public health strategies should prioritize the promotion of moderate sleep to mitigate the cognitive risks associated with aging, particularly in culturally specific contexts.

## Introduction

### Background

Cognitive impairment poses a significant challenge in the context of global aging, profoundly affecting societal development and individual well-being [1]. In China, approximately 30% of the middle-aged and older population, representing over 260 million people, experience cognitive decline [2-4], which undermines autonomy and imposes substantial economic burdens on families and society [5,6]. Among various influencing factors, sleep duration has emerged as a potential mitigator of cognitive decline, with both insufficient and excessive sleep being associated with impaired cognitive performance [7,8].

Studies conducted in the United States, such as the English Longitudinal Study of Aging (ELSA) and the Health and Retirement Study, have revealed that both short and long sleep durations adversely affect cognitive function [9,10]. Specifically, the ELSA study found that older adults who sleep less than 5 hours per night have significantly lower cognitive scores compared with those who sleep for 7 hours [11,12]. Various mechanisms have been proposed to explain these findings, including impairment of the brain’s ability to clear metabolic waste due to insufficient sleep and the reflection of underlying health problems through excessive sleep [13-15]. However, these studies are predominantly focused on Western populations, and their findings may not be entirely applicable to other cultural contexts. In China, research on the relationship between sleep duration and cognitive function is relatively scarce and often relies on single cross-sectional data [16,17]. Furthermore, systematic evaluations of this relationship, particularly with respect to sex and age differences, are lacking. In addition, most existing studies are limited to specific age groups or fail to account for the moderating effects of demographic factors such as sex and age [18]. Thus, it is crucial to conduct a more comprehensive assessment of the complex relationship between sleep duration and cognitive function.

### Objectives

Given the distinctions in historical ethnic, economic, and sociocultural background, Chinese adults had different sleep patterns contrasted with other countries [19,20]. This study uses nationally representative data from the China Health and Retirement Longitudinal Study (CHARLS) to systematically examine the relationship between sleep duration and cognitive function. By subdividing cognitive function into mental intactness and episodic memory, this study provides evidence to support improving the cognitive health among middle-aged and older people. We hypothesized that both short and longer sleep durations are strongly associated with cognitive decline.

## Methods

### Study Design

**Figure 1:**
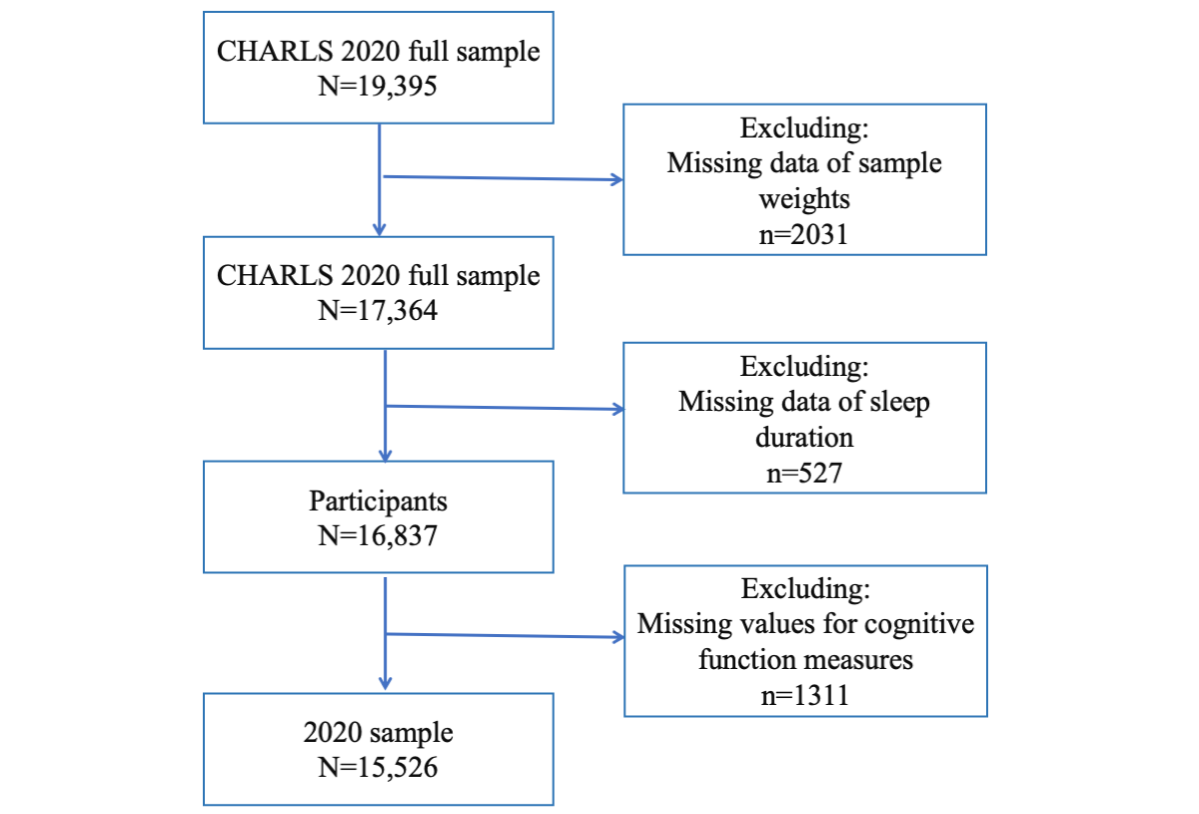
The flowchart of the study sample from the 2020 China Health and Retirement Longitudinal Study (CHARLS).

This cross-sectional study used data from the 2018 survey of CHARLS, conducted between 2018 and 2020, which surveyed residents aged 45 years or above in 28 provinces across China [21,22]. The CHARLS focused on aging-related issues in China, the largest upper-middle-income country, and aimed to align with leading international research studies to ensure cross-study comparability of results [23]. Initiated in 2011, the CHARLS conducted follow-ups every 2 or 3 years, including in 2013, 2015, 2018, and 2020, to collect a nationally representative sample of the middle-aged and older adults in China, using a multistage stratified sampling technique with 450 villages distributed in 150 counties in 28 provinces. In this study, we used the latest CHARLS wave in 2020, with 19,816 individuals, to investigate the associations between sleep duration and cognitive function. Figure 1 shows a schematic flow diagram of the study sample [24].

### Ethical Considerations

The CHARLS study protocol adhered to the principles of the Declaration of Helsinki and received ethical approval from the institutional review board of Peking University (ethical approval number IRB 00001052-11015) [25]. As this study involved a secondary analysis of existing data sets, ethical approval was not required under the research ethics policies and procedures of the London School of Economics and Political Science. This cross-sectional study follows the Strengthening the Reporting of Observational Studies in Epidemiology (STROBE) reporting guideline. All CHARLS participants provided written informed consent before data collection [26].

### Cognitive Function

To evaluate cognitive performance, we used 3 composite measures based on the framework of the Health and Retirement Study and other cognitive function research, which include the word recall test, the Telephone Interview of Cognitive Status, and a pentagon-drawing test. The word recall test assessed episodic memory by requiring respondents to immediately recall 10 Chinese nouns (immediate recall; just read to them in any order) and then recall the same list with 4-minute delay (delayed recall). The episodic memory score was calculated as the average of immediate and delayed recall scores, ranging from 0 to 10. The Telephone Interview of Cognitive Status and a pentagon drawing test were used to assess mental intactness, focusing on time orientation, numerical ability, and visuospatial skills. Participants performed tasks such as serial subtractions, identifying the current data (month, day, year, and day of the week and season) and replicating a drawing of overlapping pentagons [27,28]. The scores from these tasks were combined into a single mental intactness score, ranging from 0 to 11. The global cognitive function was calculated as the sum of the episodic memory and mental intactness scores, with higher scores indicating better cognitive performance [29,30].

### Sleep Duration

Drawing upon data from the ELSA conducted in England, CHARLS gathered self-reported sleep duration figures during face-to-face interviews using an open-ended format. Specifically, participants were queried about their average sleep hours per night over the past month: “In the past month, how many hours of sleep have you had on average per night?” In alignment with the previous CHARLS sleep study [31-34], responses were subsequently categorized into 7 sleep duration groups (≤4, 5, 6, 7, 8, 9, and ≥10 hours per evening). Daytime napping patterns were assessed through the question: “In the past month, how long have you napped on average after lunch?” leading to 4 napping duration categories: nonnappers (0 min), short nappers (<30 min), moderate nappers (30-90 min), and extended nappers (>90 min). In line with the previous CHARLS study on sleep, we divided respondents into 3 sleep duration categories in the analyses. The specific categorization is as follows: a sleep duration of 7 to 8 hours is considered the standard sleep group, less than 7 hours is considered the short sleep group, and more than 8 hours is considered the long sleep group [35].

Consistent with the approach adopted in a previous CHARLS on sleep patterns, we classified participants into 3 sleep duration categories for analysis. The detailed categorization criteria are outlined as follows: individuals with a sleep duration of 7 to 8 hours are the standard sleep group, those sleeping less than 7 hours are the short sleep group, and those with a sleep duration exceeding 8 hours are the long sleep group.

### Covariates

The CHARLS used a structured questionnaire to collect comprehensive sociodemographic and health-related data. The covariates considered potentially influential in shaping the associations included age; sex; educational attainment (elementary school and below, secondary school, and college and above); marital status (married vs others); residence (rural vs urban); smoking and drinking status (never vs current); visual and hearing impairment; self-reported general health status (ranging from very good to very poor); self-reported life satisfaction (spanning from completely satisfied to not at all satisfied); daytime napping habits; depression; and chronic disease condition (none, mild, and severe). In addition, sample weights were considered during the analysis to adjust for the representativeness of the sample.

### Statistical Analysis

In this study, ANOVA was used for numerical variables, while ordered *t* test and chi-square tests were conducted for discrete variables to compare respondent characteristics among different sleep groups, specifically short sleepers, standard sleepers, and those with long sleep disorders. Continuous variables were summarized with means and SDs, while categorical variables were reported in terms of counts and percentages. To maximize statistical power, the sample size for analysis was allowed to vary based on the quantity of valid responses for each cognitive metric. For the continuous cognitive measures, a generalized linear model with a Gaussian distribution and an identity link function was used. A comprehensive suite of covariates, encompassing demographic factors such as age, sex, educational attainment, marital status, and lifestyle attributes, including smoking and alcohol consumption, were incorporated into our analytic framework. In addition, measures of functional independence based on activities of daily living, urban versus rural residency, self-assessed health status, life satisfaction, depressive symptomatology, and the presence of chronic medical conditions were included as covariates to mitigate potential confounding effects. Statistical significance was set at 2-sided *P*<.05. All data analyses were conducted using Stata (version 18.0; Stata Corp).

## Results

### Sample Characteristics

The characteristics of the surveyed individuals are shown in Table 1. The study sample included 15,526 respondents, including 8232 female participants (53.02%) and 7294 male participants (46.98%), with an average age of 61.5 (SD 9.27) years. Among them, 7808 individuals (N=15,526, 50.29%) belonged to the standard sleep group, 6034 (N=15,526, 41.92%) to the short sleep group, and 1084 (N=15,526, 6.98%) to the long sleep group. In terms of the cognitive function composite score, participants in the standard sleep group demonstrated the highest mean score of 11.03 (SD 4.34), followed by the short sleep group with a mean score of 10.32 (SD 4.52). Conversely, the long sleep group recorded the lowest mean score of 8.40 (SD 4.67). Regarding the mental intactness composite score, a similar pattern emerged, with the standard sleep group scoring highest (mean 6.81, SD 2.96), followed by the short sleep group (mean 6.35, SD 3.06) and the long sleep group recording the lowest score (mean 5.27, SD 3.14). In the domain of episodic memory, the standard sleep group achieved a mean score of 4.22 (SD 2.06), outperforming the short sleep group (mean 3.97, SD 2.08) and the long sleep group (mean 3.13, SD 2.19). Collectively, these findings underscore the superior performance of the standard sleep group across cognitive function, mental intactness, and episodic memory domains, compared with both the short and long sleep groups. Notably, the long sleep group consistently shows the lowest scores across all 3 cognitive domains, hinting at a potential association between sleep duration and cognitive health.

**Table 1:** Characteristics of the sample by night sleep duration from the 2020 China Health and Retirement Longitudinal Study (N=15,526).

Characteristics		Total sample	Short sleep group (n=6034)	Standard sleep group (n=7808)	Long sleep group (n=1084)
Cognitive function score (grade), mean (SD)		10.42 (4.52)	10.32 (4.52)	11.03 (4.34)	8.40 (4.67)
Mental intactness score (grade), mean (SD)		6.43 (3.06)	6.35 (3.06)	6.81 (2.96)	5.27 (3.14)
Episodic memory score (grade), mean (SD)		3.99 (2.07)	3.97 (2.08)	4.22 (2.06)	3.13 (2.19)
Age (y), mean (SD)		61.50 (9.27)	61.79 (9.16)	60.34 (9.20)	64.54 (9.75)
**Sex, n (%)**					
	Male	7294 (46.98)	4124 (56.54)	2622 (35.95)	548 (7.5)
	Female	8232 (53.02)	2510 (30.49)	5186 (63.00)	536 (6.51)
ADL^a^ impairment, n (%)		3389 (21.83)	2393 (70.61)	768 (22.66)	228 (6.73)
Normal ADL, n (%)		12,137 (78.17)	6917 (56.99)	4364 (35.96)	856 (7.05)
IADL^b^ impairment, n (%)		2957 (19.05)	2057 (69.56)	663 (22.42)	237 (8.01)
Normal IADL, n (%)		12,569 (80.95)	7253 (57.71)	4469 (35.56)	847 (6.74)
**Education, n (%)**					
	Illiterate	3341 (21.52)	2037 (60.97)	934 (27.96)	370 (11.07)
	Middle school	10.28 (66.21)	6143 (59.76)	3.49 (33.91)	651 (6.33)
	High school	1905 (12.27)	1130 (59.32)	712 (37.38)	63 (3.31)
Married, n (%)		11,977 (77.14)	6981 (58.29)	4161 (34.74)	835 (6.97)
Smoke, n (%)		4018 (25.88)	7035 (61.13)	3713 (32.26)	760 (6.60)
Drink use, n (%)		5638 (36.31)	3342 (59.28)	1960 (34.76)	336 (5.96)
Rural areas, n (%)		11,794 (75.96)	6964 (59.05)	3732 (32.9)	942 (7.99)
Depressed, n (%)		6123 (39.44)	4324 (70.62)	1432 (23.39)	367 (5.99)
Feeling of own good health, n (%)		3734 (24.05)	1805 (48.34)	1602 (42.90)	327 (8.76)
Subjective well-being, n (%)		13,810 (88.95)	8069 (58.43)	4762 (34.48)	979 (7.09)
**Chronic diseases, n (%)**					
	None	9936 (64.00)	5793 (58.30)	3458 (34.80)	685 (6.89)
	Single chronic disease	5026 (32.37)	3126 (62.20)	1530 (30.44)	370 (7.36)
	Multiple chronic diseases	564 (3.63)	391 (69.33)	144 (25.53)	29 (5.14)

^a^ADL: activities of daily living.

^b^IADL: instrumental activities of daily living; defined as having a score of 10 or above on the 10-item Center for Epidemiologic Studies Depression scale.

### Trajectories for Sleep Duration Across Cognition Status

**Figure 2:**
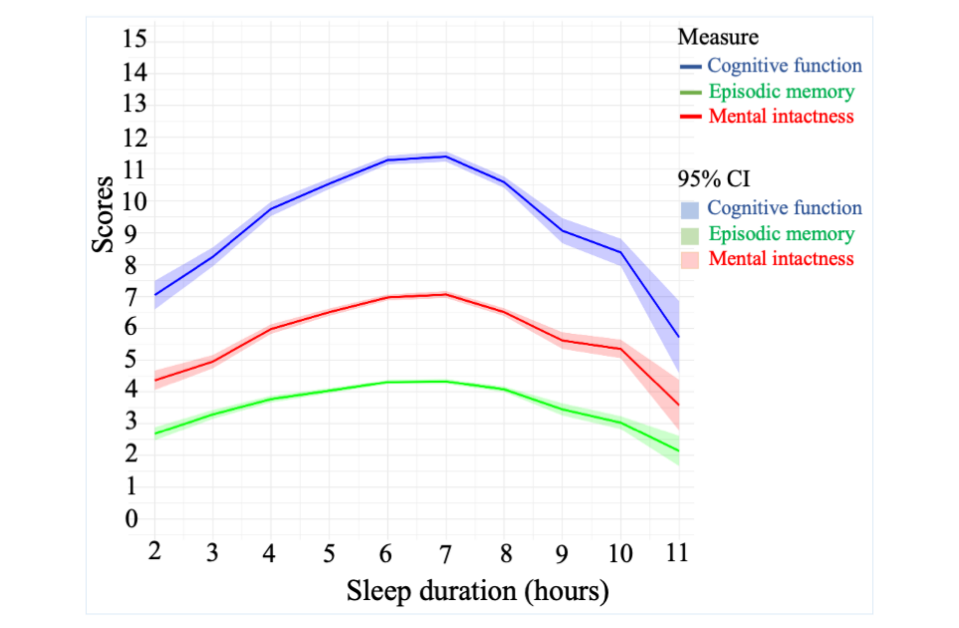
Trajectories of cognitive scores across groups with different sleep durations.

Figure 2 displays the trajectories of cognitive scores across different sleep durations, including cognitive function, mental intactness, and episodic memory. Overall, in 2020, the relationship between sleep duration and the scores of the selected population in cognitive function, mental intactness, and episodic memory exhibited an inverse U-shaped pattern.

In Table 2, in the unadjusted analysis, compared with individuals sleeping for 7 hours, those in the long sleep group (≥10 hours) exhibited the most significant negative impact on cognitive function (β=−1.85, 95% CI −2.17 to −1.52; *P*<.001). Subsequently, the 9-hour sleep group demonstrated the second-highest negative effect with β=−0.85 (95% CI −1.02 to −0.64; *P*<.001). Unhealthy sleep patterns had a detrimental influence on mental intactness than on episodic memory. Specifically, in the very short sleep group (≤4 hours), the negative effect on mental intactness was β=−0.61, whereas for episodic memory, it was β=−0.22. Intriguingly, no significant association was observed between cognitive function, episodic memory, and mental intactness with sleep duration in the 6-hour sleep group (*P*>.05). Upon adjustment, the long sleep group (≥10 hours) showed an exacerbated negative impact on overall cognitive function, with β increasing from −1.85 to −3.01, 95% CI −3.39 to −2.63; *P*<.001). The second most significant negative effect shifted from the 9-hour sleep group to the very short sleep group (≤4 hours) in the adjusted model with β=−1.85 (95% CI −2.07 to −1.62; *P*<.001). Consistent with the unadjusted findings, unhealthy sleep continued to have a greater adverse effect on mental intactness compared with episodic memory. The 6-hour sleep group maintained a nonsignificant association with cognitive function (*P*>.05).

**Table 2:** Associations between sleep duration and cognitive function in participants from the 2020 China Health and Retirement Longitudinal Study.

Model and sleep duration (hr)		Cognitive function		Episodic memory		Mental intactness	
		β	95% CI	β	95% CI	β	95% CI
**Model 1^a^**							
	≤4	−0.83	−1.02 to −0.64	−0.22	−0.32 to −0.12	−0.61	−0.75 to −0.48
	5	−0.24	−0.43 to −0.05	−0.03	−0.13 to −0.06	−0.21	−0.34 to −0.07
	6	−0.08	−0.25 to −0.10	0.01	−0.08 to −0.10	−0.09	−0.21 to −0.03
	7	1 (reference)	1 (reference)	1 (reference)	1 (reference)	1 (reference)	1 (reference)
	8	−0.45	−0.64 to −0.25	−0.09	−0.19 to −0.01	−0.36	−0.49 to −0.22
	9	−0.85	−1.18 to −0.52	−0.29	−0.46 to −0.13	−0.56	−0.79 to −0.33
	≥10	−1.85	−2.17 to −1.52	−0.74	−0.91 to −0.57	−1.11	−1.34 to −0.88
**Model 2^b^**							
	≤4	−1.85	−2.07 to −1.62	−0.58	−0.69 to −0.48	−1.26	−1.42 to −1.11
	5	−0.55	−0.78 to −0.33	−0.16	−0.26 to −0.05	−0.40	−0.55 to −0.25
	6	−0.12	−0.33 to −0.08	−0.01	−0.10 to −0.09	−0.11	−0.26 to −0.03
	7	1 (reference)	1 (reference)	1 (reference)	1 (reference)	1 (reference)	1 (reference)
	8	−0.79	−1.01 to −0.56	−0.21	−0.32 to −0.10	−0.57	−0.73 to −0.42
	9	−1.78	−2.17 to −1.39	−0.61	−0.79 to −0.43	−1.17	−1.43 to −0.90
	≥10	−3.01	−3.39 to −2.63	−1.15	−1.33 to −0.97	−1.86	−2.13 to −1.60

^a^Model 1 was unadjusted.

^b^Model 2 was adjusted for activities of daily living, instrumental activities of daily living, education, marital status, smoking, alcohol consumption, residence, self-rated health, self-rated life satisfaction, depression, chronic diseases condition, and sampling weights.

## Discussion

### Principal Findings

This nationally representative study, using the CHARLS database, reveals a statistically significant inverse U-shaped association between sleep duration and cognitive function. The findings reveal that the standard sleep group had the optimal cognitive scores, episodic memory, and mental intactness, whereas groups with either short or long sleep durations were significantly associated with cognitive decline, with long sleep having a more pronounced negative effect.

These findings align with those from major international studies, such as the ELSA and the Health and Retirement Study, which also identified the detrimental effects of both short and long sleep on cognitive function [12,36]. The consistent inverted U-shaped relationship between sleep duration and cognition suggests a universal phenomenon, albeit influenced by cultural and social contexts. However, it is important to recognize that cultural and social contexts may partly explain the observed variations in findings across different populations. For instance, in Chinese culture, familial support and older adult lifestyles may exacerbate the negative effects of prolonged sleep duration. The emphasis on close family bonds in Chinese society could inadvertently lead to excessive care or protection of older adults [37], potentially reducing their physical activity levels and contributing to cognitive decline. In addition, traditional health beliefs that promote prolonged rest as beneficial may further amplify the prevalence of long sleep among older adults in China [38]. These factors, coupled with a slower pace of life and lower levels of social engagement in some Chinese populations, present a unique cultural framework that differs from Western populations, where long sleep durations are less prevalent [39]. Thus, the findings of this study impart profound insights into how cultural and social factors influence the interplay between sleep duration and cognitive function, offering perspectives that are infrequently explored in the realm of international scholarly research.

From a mechanistic perspective, the adverse consequences of short sleep on cognitive function are well-documented. Short sleep may hinder the elimination of cerebral metabolic waste, including β-amyloid, a neurotoxic protein linked to Alzheimer disease [40,41]. Inefficient removal of such waste materials can disrupt neuronal function and impair cognitive processes. Moreover, sleep deprivation interferes with the equilibrium of neurotransmitters, including dopamine and serotonin, which are critical for mood regulation, attention, and decision-making [13]. These disruptions can culminate in impairments in executive function and emotional regulation, both integral aspects of mental well-being. Furthermore, hormonal dysregulation associated with insufficient sleep, such as elevated cortisol levels, has been shown to exert neurotoxic effects on the hippocampus, an essential brain region for memory consolidation. Moreover, decreased levels of growth hormone during sleep deprivation may also hinder tissue repair and neuronal regeneration, further compromising brain health [42].

The severity of short sleep’s impact varies across different cognitive domains. Mental integrity, along with episodic memory, suffers from sleep deprivation; however, the disruptions to mental integrity appear more profound and systemic. Insufficient sleep not only impairs attention, executive function, and emotional stability but also exerts broad effects on mental health and cognitive capabilities, mediated by chronic stress and heightened inflammatory processes, ultimately leading to structural damage in the prefrontal cortex and neurotransmitter imbalances [43]. Conversely, while the impact on episodic memory is notable, it is relatively confined to specific processes such as memory formation and consolidation, often stemming from hippocampal dysfunction and increased susceptibility to memory interference. Therefore, the systemic nature of sleep deprivation’s effects on mental integrity renders then more comprehensive and extensive when compared with its effects on episodic memory [44]. In contrast, long sleep duration is frequently associated with underlying health conditions that may indirectly contribute to cognitive decline. For instance, prolonged sleep has been linked to elevated inflammatory markers like C-reactive protein and metabolic dysregulation [45,46]. These biological responses could reflect the presence of chronic illnesses or suboptimal health states adversely affecting cognitive function. In addition, sex differences in physiological, hormonal, and social factors may further exacerbate cognitive disparities associated with long sleep [47,48]. Specifically, variations in estrogen levels and their neuroprotective effects could partly account for differing cognitive outcomes between men and women. Socially, women may encounter distinct roles and stressors that interact with sleep patterns, potentially influencing cognitive health in unique ways [49]. These findings highlight the need for a sophisticated appreciation of the interplay between biological, social, and sex-specific factors with sleep duration in influencing cognitive outcomes.

Since the declines are primarily centered on mental intactness, the resulting downstream consequences on instrumental activities of daily living warrant explicit consideration, particularly for older adults living alone. Low scores in these domains map onto tangible challenges in everyday life. Impairments in mental intactness, encompassing attention, and sequencing and visuospatial copying abilities, are closely tied to instrumental activities of daily living, which include managing medications and finances, keeping appointments, using the phone, navigating public transport, or following multistep cooking and home-safety routines. Deficits in episodic memory further undermine an individual’s capacity for new learning and recall. This, in return, affects their adherence to treatment plans, remember emergency contacts, or recall whether gas or electric appliances have been turned off. Together, these impairments heighten risks that go far beyond a mere reduction in independence, which includes medication errors, missed or duplicate doses, falls resulting from disorientation, wandering or getting lost outdoors, malnutrition due to irregular meals, fire hazards, and vulnerability to financial scams. These risks are particularly pronounced among older adults living alone, underscoring the need for proactively identifying at-risk sleepers at both the clinical and community levels.

The study’s strengths lie in its use of nationally representative data from the CHARLS, bolstering the external validity and generalizability of the findings. The substantial sample size reinforces the robustness of the results, enabling an in-depth analysis of sleep duration and its correlation with cognitive function among middle-aged and older adults. Importantly, this research contributes significant scientific evidence to inform sleep-based interventions aimed at promoting cognitive health. Translating these strengths into action, we outline implementable strategies for prediction, public communication, and prevention that public-sector and primary-care services can adopt now.

These findings provide a solid foundation for developing predictive models. They motivate nonlinear specifications (eg, restricted cubic splines or categorical cut-points at ≤4, 5, 6, 7 [reference], 8, 9, and ≥10 hours) combined with demographic, health, and psychosocial covariates already collected in population surveys and primary care. Models can be trained to estimate individual risk of low global cognition and domain-specific deficits (especially attentional or executive components captured by mental intactness), enabling risk stratification, providing early warning signals for clinicians, and targeting of behavioral counseling for those who stand to gain the most.

The study also offers data to support public awareness initiatives. Given the clear, nonlinear risk signal at both extremes of sleep duration and the relative sparing at approximately 7 hours, public health messaging can be framed in plain language (eg, “both insufficient and excessive sleep are linked with worse thinking and attention in later life”), while emphasizing that attention or executive skills appear especially vulnerable to chronic unhealthy sleep habits.

While acknowledging its strengths, several limitations merit recognition. First, the cross-sectional design of this study precludes the establishment of causal relationships between sleep duration and cognitive function, necessitating longitudinal studies to ascertain whether sleep duration directly influences cognitive trajectories over time or if other factors mediate this relationship. Second, the reliance on self-reported sleep data may introduce information bias, potentially affecting the accuracy and reliability of the results. Incorporation of objective sleep measures, such as actigraphy or polysomnography, could yield more precise assessments in future studies. Third, the relatively smaller sample size in older age groups may have contributed to increased variability in the findings, diminishing the statistical power to detect significant associations in this subgroup. Finally, the study did not account for other potential confounding factors, such as dietary habits, genetic predispositions, or environmental influences, which may play crucial roles in shaping the observed relationships and warrant further exploration.

In prioritizing future research endeavors, longitudinal study designs should be used to confirm causal relationships and examine potential mediators and moderators of the relationship between sleep duration and cognitive function. For instance, biomarkers indicative of inflammation and metabolic health could provide valuable insights into the underlying biological mechanisms. Similarly, lifestyle variables, including physical activity, social engagement, and dietary habits, could elucidate the broader context of sleep-cognition interactions. In addition, comparative analyses across diverse cultural milieux could augment our comprehension of how sociocultural factors shape these relationships, ultimately guiding the development of tailored interventions to foster cognitive health in various populations.

### Conclusions

In summary, this study reveals that moderate sleep duration is significantly protective of cognitive function in middle-aged and older adults. The identified risks associated with both short and long sleep underscore the necessity for individualized sleep health interventions tailored to personal characteristics. Public health policies should account for sex and age disparities to implement effective interventions aimed at enhancing cognitive health in aging populations, thereby alleviating the societal and economic burdens associated with global aging.

## Abbreviations

CHARLS: China Health and Retirement Longitudinal Study
ELSA: English Longitudinal Study of Aging
STROBE: Strengthening the Reporting of Observational Studies in Epidemiology
